# Differential admixture in Latin American populations and its impact on the study of colorectal cancer

**DOI:** 10.1590/1678-4685-GMB-2020-0143

**Published:** 2020-11-13

**Authors:** Valentina Colistro, Patricia Mut, Pedro C. Hidalgo, Angel Carracedo, Inés Quintela, Augusto Rojas-Martínez, Mónica Sans

**Affiliations:** 1 Universidad de la República, Facultad de Medicina, Departamento de Métodos Cuantitativos, Montevideo, Uruguay.; 2 Universidad de la República, Facultad de Humanidades y Ciencias de la Educación, Departamento de Antropología Biológica, Montevideo, Uruguay.; 3 Universidad de la República, Centro Universitario de Tacuarembó, Polo de Desarrollo Universitario Diversidad Genética Humana, Tacuarembó, Uruguay.; 4 Universidad de Santiago de Compostela, Centro Nacional de Genotipado (CEGEN), Spain.; 5 Universidade de Santiago de Compostela, CIBER de Enfermedades Raras (CIBERER)-Instituto de Salud Carlos III, Grupo de Medicina Xenómica, Santiago de Compostela, Spain.; 6 Escuela de Medicina y Ciencias de la Salud, Tecnológico de Monterrey, Monterrey, México.

**Keywords:** Admixture, genetic ancestry, heterozygosity, Latin American populations

## Abstract

Genome-wide association studies focused on searching genes responsible for several diseases. Admixture mapping studies proposed a more efficient alternative capable of detecting polymorphisms contributing with a small effect on the disease risk. This method focuses on the higher values of linkage disequilibrium in admixed populations. To test this, we analyzed 10 genomic regions previously defined as related with colorectal cancer among nine populations and studied the variation pattern of haplotypic structures and heterozygosity values on seven categories of SNPs. Both analyses showed differences among chromosomal regions and studied populations. Admixed Latin-American samples generally show intermediate values. Heterozygosity of the SNPs grouped in categories varies more in each gene than in each population. African related populations have more blocks per chromosomal region, coherently with their antiquity. In sum, some similarities were found among Latin American populations, but each chromosomal region showed a particular behavior, despite the fact that the study refers to genes and regions related with one particular complex disease. This study strongly suggests the necessity of developing statistical methods to deal with di- or tri-hybrid populations, as well as to carefully analyze the different historic and demographic scenarios, and the different characteristics of particular chromosomal regions and evolutionary forces.

## Introduction

One of the greatest challenges in genetic epidemiology is the development and application of methodological strategies allowing identification of genetic risk loci in order to achieve a more thorough understanding of the genetic basis of complex diseases, as they are the result of interactions between multiple genetic and/or environmental factors, each with modest effects. It is likely that different combinations produce the same clinical symptoms (Botstein and Risch, 2003). Also, many complex diseases are genetically related, sharing common genetic risk variants (Teng *et al.*, 2016). Moreover, interconnections among all genes expressed in disease-relevant cells and the core disease-related genes (“omnigenic” model) (Boyle *et al.*, 2017).

Linkage and association studies are the two main approaches applied to identify the genetic basis of these types of diseases (Morton, 2003; Patel *et al.*, 2003; Khoury *et al.*, 2010). Linkage studies are more efficient in detecting genes with large effects, like single-gene based disorders, but they lack the statistical power to detect variants with modest effects. On the other hand, genome-wide association studies (GWAS) have a statistical advantage as they provide greater power for detecting common variants with modest risk (Risch and Merikangas, 1996). However, these studies have been criticized, as they rely on an extremely high number of markers in order to be carried out (more than 100.000), a large quantity of samples, as well as adequate technological resources to process the enormous amount of data, becoming impractical and very expensive (Cantor *et al.*, 2010; Qin and Zhu, 2012).

Admixture mapping studies (AMSs) constitute an alternative approach. This methodology was first proposed by Rife (1953), but its implementation has been technically possible only in the last decades (McKeigue, 2005). AMS is based on the gene flow processes between continental populations occurring in the last centuries, producing particular chromosome configurations in the resulting admixed populations, showing a mosaic of ancestry segments (Darvasi and Shifman, 2005). When a disease has substantial despair prevalence among parental populations, the risk allele locus will show an over-representation ancestry of the high risk population in the admixed population. The use of ancestry informative markers (AIMs) allows the identification of the population source of the studied chromosomal segments (Tian *et al.*, 2008; Winkler *et al.*, 2010, among others). The effect of rare variants in recently admixed populations can be much greater compared with its ancestral populations, as has been shown by Moltke and Albrechtsen (2014). Moreover, the effects of noncausal genetic variants depend on its correlation with causal variants, and these last may vary depending on the ancestral populations and the patterns of linkage disequilibrium (Skotte *et al.*, 2019).

The process of admixture in the Americas can be seen as a natural experiment for genetic epidemiology and anthropology, in which polymorphic marker loci are used to infer a genetic basis for traits of interest (Chakraborty and Weiss, 1988). Nowadays it is possible to establish a maximum of approximately 21 generations of admixing, depending on the region. Cosmopolitan Latin American populations have Native contributions from around 1% to more than 50%, and African contributions from 2 to 40%, while on the other side, it is rare to find Native groups without any admixture (Sans, 2000).

The grade of contribution of each parental population will be reflected not only in the amount of chromosomes from each ancestral origin, but in the quantity of blocks from these origins inside chromosomes, and depend on the admixture process (Pfaff *et al.*, 2001). We assume that the antiquity of populations is directly related to the heterozygosity and the size of chromosomal blocks; consequently, we expect smaller blocks in more ancient populations. Moreover, heterozygosity can be related to the time (generations) after a process of admixture, assuming that non-admixed populations are more homogeneous. We recognize that it is an oversimplification because it ignores the microevolutionary changes in the admixed population, as genetic drift, selection and gene flow.

The major aim of our study was to understand the process that generates complex chromosome patterns in admixed populations and to improve the implementation of AMSs in Latin American populations. Particularly, this study was focused on analyzing genes and chromosomal regions previously related to colorectal cancer (CRC) in admixed populations, because past studies were mainly based on populations of European descent. CRC is common in both sexes and has no major avoidable risk factor. By determining the ancestral proportions, as well as the heterozygosity and size of fragments in five admixed American populations and several populations from Europe, Africa and Asia in associated regions, we intend to help in the understanding of genetic CRC causes.

## Subjects and Methods

### Samples

We used data available in 1000 Genomes Project for eight populations and an unpublished set of genetically admixed Mexican samples. Regarding the 1000 Genome Project samples (The 1000 Genomes Consortium, 2010), five are admixed populations from the Americas, and the others were selected to represent part of their parental populations. The admixed populations were: Afro-Americans from the United States (ASW, N=83), Colombians (CLM, N=60), Puerto Ricans (PUR, N=55), Peruvians (PEL, N=85) and Mexicans from Los Angeles, CA (MXL, N=76). The samples from Africa, Europe and Asia were selected due to their relationship to the migrations toward America, being the last ones considered in substitution of Native Americans. We are aware of differences between Asian and Native American populations, but we choose this alternative due to the scarcity of data referred to the SNPs and regions considered for such populations. Therefore, we analyzed Yorubas and Luhya to represent African populations (denominated Africans, AF, in this study, N=176), Iberians, Tuscans, and Utah residents with northern and western European ancestry for European populations (denominated EU, N=174), and Chinese from Beijing, Southern Han Chinese and Japanese from Tokyo to represent Asians (denominated AS, N=98).

We are particularly interested in another Mexican sample (hereafter, MEX, N=831) because it is formed by healthy controls of a GWAS study of CRC (CHIBCHA, study of hereditary cancer in Europe and Latin America). The individuals were recruited in different blood banks, three in Mexico City (Centro Médico Nacional Siglo XXI of the Mexican Social Security Institute -IMSS), three in Monterrey (UMAE 25, IMSS and the University Hospital of the Universidad Autónoma de Nuevo León) and three in Torreon (UFM 16 IMSS, the UMAE 71 IMSS, and the University Hospital of Torreon), from 2010 to 2012. All subjects gave informed consent for inclusion before they participated in the study. The protocol was approved by the ethics committees of each participating institution (Ethics Committee of the University Hospital, Universidad Autónoma de Nuevo León code BI10-003 and the National Commission of Scientific Research of the Mexican Social Security Institute code R-2012-785-032), the Federal Commission for Protection against Health Risks (COFEPRIS), code CMN2012-001, and the Ethics Committee of CHIBCHA project number: 223 678.

Samples were genotyped using two complementary arrays: Axiom Genome-Wide LAT 1 (Latino) Array and a Custom-designed Array, both from Affymetrix Axiom Genotyping Solutions. The former was designed to maximize coverage of common and rare disease-associated alleles in Latin American populations that have genetic contributions from European, Native American and African ancestries. The latter was specifically designed for this study, being the SNPs selection based on regions previously detected as associated with CRC. SNP calling in both arrays was done following Affymetrix best practice workflow, which includes the Genotyping Console Software in combination with SNPolisher. A total of 1,169,944 SNPs (387,948 from the Custom Array and 781,996 from the Latino Array) was obtained. These samples were included because its large number of individuals and the high coverage of SNPs in the considered regions represent an opportunity to compare the performance of another admixed population.

Genotypes of Native American (NAM) samples were used in order to estimate the global individual ancestry. These genotypes included individuals from five ethnic groups: Zapotecs from Oaxaca, Mexico (N=21), Tepehuans from Durango in Northern Mexico (N=23), Nahuas from Central Mexico (N=14), Mayas from Campeche, Mexico (N=25), Quechuas from Cerro de Pasco, Perú (N=24) and Aymaras from La Paz, Bolivia (N=25). We consider a panel of AIMs developed and optimized for the study of Latin American populations by the LACE Consortium (for detailed information about the panel and the populations refer to Galanter *et al.*, 2012). This panel was composed of 446 AIMs but the ancestry analysis performed in the present study was limited to the 275 SNPs shared with the Mexicans, the 1000G populations and the Native American samples.

### Genomic regions studied

We selected 10 autosomal regions, with an average size of 680.9 Kbp spanning a total of 6.8 MB (Table 1). These regions were previously described to show association to CRC (Kinzler *et al.*, 1991; Aaltonen *et al.*, 2007), seven of them are genes: *APC*, *BRAF*, *MSH2*, *MSH6*, *MLH1*, *MUTYH* and *PMS2*, and three are loci described by Carvajal-Carmona *et al.* (2011) also associated with CRC: 8q23.3, 16q22.1 and 19q13.11.

**Table 1:** The 10 genomic regions considered in the analysis, 7 were genes and 3 were locations (± 1 MB). The table shows chromosome, base pair start and end, gene name, cytoband and number of SNPs of each studied location.

Chromosome	Band	Gene Start (bp)	Gene End (bp)	Gene Name	SNPs
1	p34.1	45794835	45806142	MUTYH	706
2	p21	47630108	47789450	MSH2	1058
2	p16.3	48010221	48034092	MSH6	1011
3	p22.2	37034823	37107380	MLH1	997
5	q22.2	112043195	112181936	APC	1140
7	q34	140424943	140624564	BRAF	1138
7	p22.1	6012870	6048756	PMS2	682
8	q23.3	116631278	118626279	——	864
16	q22.1	67824395	69816284	——	964
19	q13.11	32534093	34530086	——	1025
Total					9585

For the seven gene regions, SNPs within the gene limits were retrieved, and in the three other regions, 1 MB upstream and downstream SNPs were considered. The number of available SNPs in each region is listed in Table 1.

### Admixture analysis

In order to understand the structure of the MEX sample, we performed a global individual admixture analysis using the AIMs panel described above. Estimation of individual admixture fractions were calculated with ADMIXTURE software version 1.3.1 (Alexander *et al.*, 2009), which considers a likelihood model. To choose the correct value of k we computed the cross-validation error over k, from 2 to 6. We found that k=3 yielded the lowest cross-validation error (k_3_=0.538) compared to other k values (k_2_=0.63968, k_4_=0.54016, k_5_=0.54226 and k_6_=0.542).

Complementary, we also analyzed the mean population admixture in each of the 10 regions for the admixed populations. In this case we were not able to use the Native American samples due to their limited number of SNPs yielding at these 10 regions. As explained above, we used the Asian samples instead. A total of 5283 SNPs were used for this analysis.

### Analysis of genetic variation

The genetic variation analysis was performed only on the seven genomic regions corresponding to genes. To compare the variation in the studied regions among the nine populations, we considered two measures using PLINK version 1.9 (Purcell *et al.*, 2007; Chang *et al.*, 2015): heterozygosity and haplotypic structures among regions and populations.

For the heterozygosity determination, the mean values of heterozygosity were analyzed for each gene by population and the mean values of SNPs were classified in seven categories. The SNPs classification categories are related to their position and consequence to transcript and were obtained using Biomart (Haider *et al.*, 2009): intronic, non-synonymous coding, synonymous coding, 5’ UTR, 3’ UTR, stop gained and stop lost.

Inference of haplotype phase was determined with the Beagle software version 4 (Browning and Browning, 2007). Gabriel *et al.* (2002) criteria were followed to define haploblocks. The allelic association between pairs of SNPs was measured by the D’ parameter (Lewontin, 1964). The distribution of blocks length (in bp) among populations was compared. Linkage analysis and haploblock estimation were done using PLINK version 1.9 (Purcell *et al.*, 2007) *.*

## Results

### Admixture analysis

**Figure 1:**
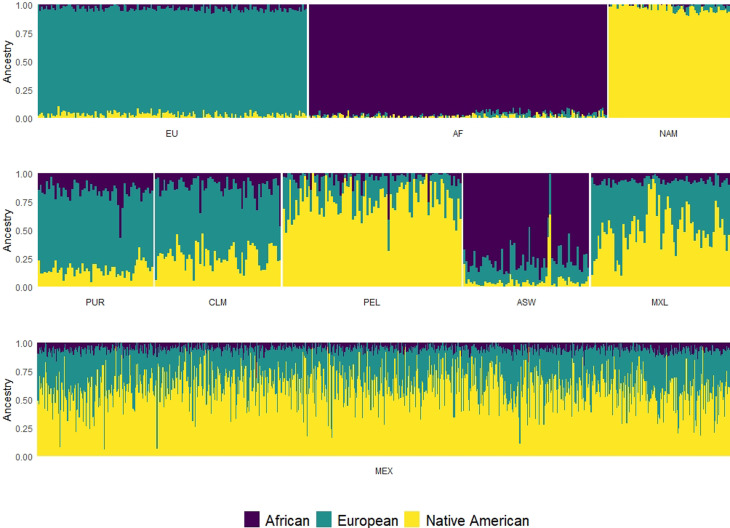
Global admixture analysis performed in ADMIXTURE, with k=3 representing the 3 ancestral components of the Admixed American populations. The barplots show each individual as a vertical line, and the ancestries are indicated by different color (NAM= Native American ancestry, AFR= African ancestry and EUR= European ancestry). a) Parental populations, b) Admixed populations from 1000G and c) Mexican unpublished samples.

The AIM panel accurately discriminates parental populations, as can be seen in Figure 1a. The representation of the global individual ancestral fractions for the admixed populations is shown in Figure 1b and 1c. According to the estimations, the ASW population has 75,4% of African ancestry, while the African proportions for the other admixed populations were lower: 12,1% in CLM, 6,8% in MXL, 4,3% in PEL and 16% in PUR. Peruvian samples (PEL) have the highest proportions of Native American ancestry (77,1%) followed by the Mexican samples (MXL and MEX) (51,2 and 61,5%, respectively). The European ancestry has its maximum in Puerto Rico (68,7%) followed by the Colombian sample (61,7%) (Table 2).

**Table 2:** Mean values of heterozygosity by population and by region. Highest and lowest values of each row were marked in order to facilitate visualization.

Gene	AF	ASW	AS	EU	PUR	CLM	PEL	MEX	MXL
APC	0,063	0,066*	0,046	0,056	0,049	0,047	0,0378†	0,044	0,044
BRAF	0,079*	0,080	0,046	0,036	0,048	0,041	0,031	0,027†	0,029
MLH1	0,080	0,094*	0,016†	0,059	0,052	0,052	0,052	0,057	0,061
MSH2	0,063	0,065*	0,049†	0,052	0,054	0,061	0,052	0,052	0,054
MSH6	0,053	0,051	0,025†	0,082*	0,079	0,078	0,035	0,047	0,049
MUTYH	0,031	0,026	0,039*	0,024	0,032	0,032	0,020†	0,026	0,028
PMS2	0,094	0,101*	0,079	0,080	0,080	0,071	0,077	0,075	0,068†
Total	0,071	0,073*	0,045	0,052	0,054	0,051	0,042†	0,044	0,045

* highest value in row;

† lowest value in row

**Figure 2:**
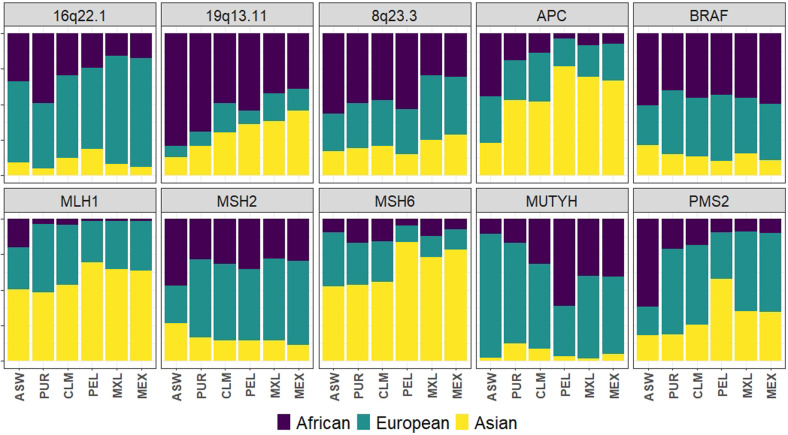
Admixture analysis by region performed with ADMIXTURE, with k=3 representing the 3 ancestral components of the Admixed American populations. The barplots show the mean ancestry of each population, and the ancestry proportions are indicated by different colors.

When the admixture analysis was performed on those 10 regions considered in this study, the results show high variability (Figure 2). No clear pattern is detected among the different regions. In general terms, there is a greater concordance among populations than among genes and regions. The greatest similarity is between both Mexican samples, while Peruvians seems to be the most dissimilar. While in MSH6 and MLH1 genes, a greater contribution of Asian ancestry was detected, and in 16q22.1 and MUTYH the European contribution is the highest.

### Genetic variation

**Figure 3:**
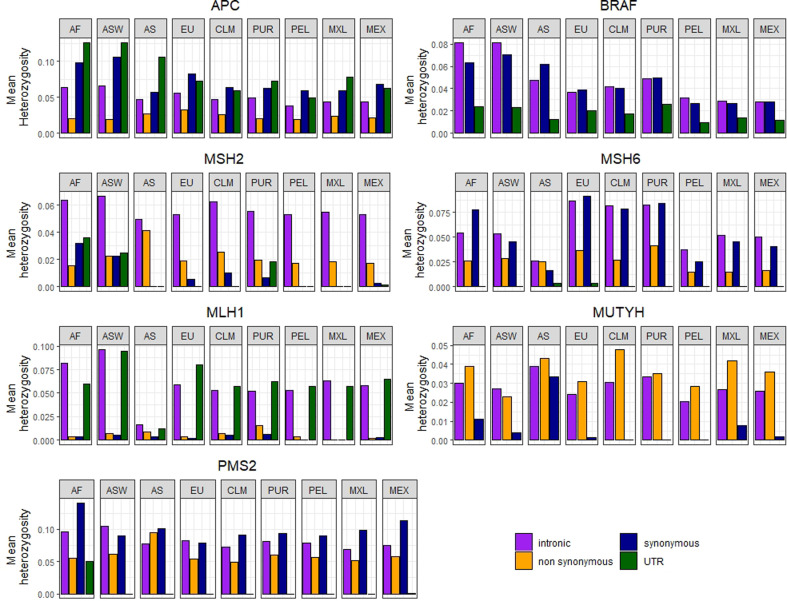
Mean heterozygosity values in four SNPs categories by gene and population. Each bar corresponds to a SNP category in a certain gene and population. SNPs categories are: intronic, non-synonymous, synonymous and UTR.

The results of the analyses of the mean heterozygosity by gene are shown in Table 2 and the mean heterozygosity using the categories of SNPs mentioned above are shown in Figure 3. For two of these categories (stop gained, stop lost), no population showed heterozygosity in any region.

The greatest mean values of heterozygosity for most of the genes are found in the ASW, except for BRAF, MSH6 and MUTYH where the greatest values are in AF, EU and AS respectively. And the lowest values are found in AS for MLH1, MSH2 and MSH6; in PEL for APC and MUTYH, in MEX for BRAF and in MXL for PMS2 (Table 2).

When including the SNP category in the analysis, different genes show different situations: a) heterozygosity related to categories of SNPs vary in different regions; b) some chromosomal regions do not show heterozygosity in some categories of SNPs; c) heterozygosity varies when considering different populations, but its behavior is relatively coherent in the different categories: Africans and Afro-descendants, European and Asian, and Latin American admixed ones.

For example, for the *APC* gene, relative differences in heterozygosity values associated to the categories of SNPs remain constant in all populations being 3’UTR the one with greatest values of heterozygosity followed by synonymous variants, except in the EU and CLM samples, where synonymous variants are greater (Figure 3).

Regarding the *BRAF* gene, the diversity among populations is clear. For this gene, the African related samples (AFR and ASW) have higher values of heterozygosity in intronic and synonymous categories, while 3’UTR regions are more homogeneous. Puerto Rico has an intermediate place between African related and other considered populations (Figure 3).

The *MSH2* locus differs from the others analyzed. The 3’UTR SNPs show none or very small heterozygosity in every population, except for the AF, ASW and PUR samples. As populations and MXL do not have heterozygosity in 3’UTR and synonymous mutations, while MEX shows very little heterozygosity in those regions. Intronic SNPs show the higher heterozygosity in every population (Figure 3).

It is important to note that the admixed Latin-American samples (PEL, CLM, MXL, MEX and PUR) show heterozygosity values for all genes that tend to be intermediate among the values of the parental samples (EU, AS and AF). Although, the ASW, also admixed, shows a pattern closer to the AF than to any other sample, in concordances with the high contributions of African genes (76%); in some cases, also PUR approximates more to those samples.

**Figure 4:**
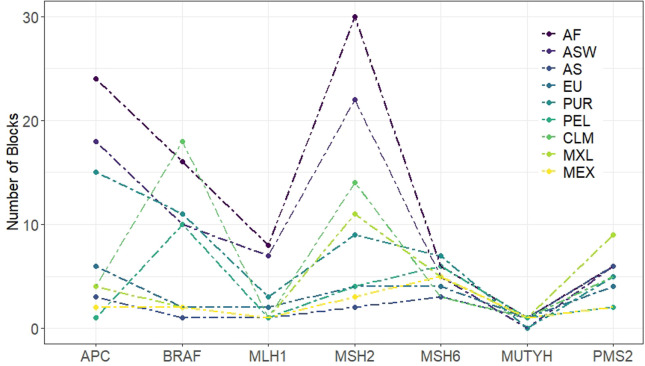
The graphic shows the number of phased haplotype blocks estimated for the 7 genes detailed per population.

The quantity of phased haplotype blocks per gene was analyzed for each population (Figure 4). The African populations (AF and ASW) have more blocks per region for most of the genes, while Asians (AS) have fewer, followed by MXL, probably because of the high Native American contribution of Native genes (62%), and by Europeans. All populations have a similar curve for the 7 genes, with some exceptions: CLM shows a large amount of haplotype blocks in *BRAF*, PUR that shows more blocks in *MSH6*, and MEX that shows more blocks at *MSH2* and *PMS2* genes and an unexpected behavior related to the other Mexican sample (MXL).

**Figure 5:**
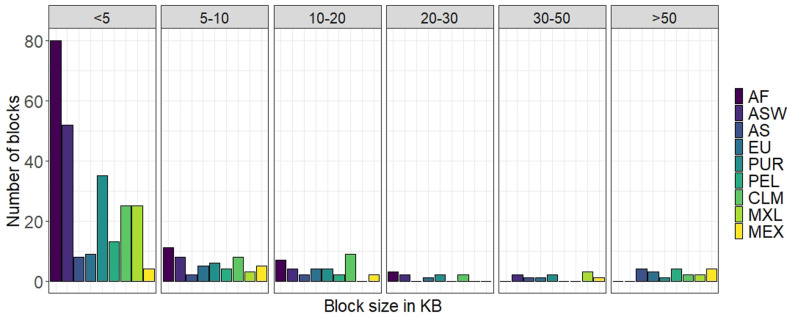
Block characteristics size (in kb) distribution of all haplotype blocks found in the analysis. Summary of haplotype diversity across all blocks.

The variability of the size of the blocks shows diversity among populations (Figure 5). It varied from < 1 kb to over 190 kb, though most of the blocks were small (< 5 kb). Markedly the African related populations (AF and ASW) have higher proportions of small blocks, and the admixed populations (CLM, MXL, MEX and PUR) are placed in an intermediate value between the African-related and the other two parental populations (AS and EU). In the MEX sample, the smaller blocks are underrepresented in comparison with the rest of the admixed samples, while they show a greater number of longer blocks related to the rest of admixed populations.

## Discussion

The results obtained using the selected AIMs supports the use of these markers for detecting admixture in Latin American populations, as demonstrated in several studies performed before (Mao *et al.*, 2007; Halder *et al.*, 2008; Tian *et al.*, 2008; Silva *et al.*, 2010; Galanter *et al.*, 2012; Manta *et al.*, 2013). Moreover, we found that the expected proportions of ancestry are consistent with the historical and geographical affinities of the samples used, as well as other estimations (Norris *et al.*, 2018). Peruvian and both Mexican samples showed the highest Native contribution, being 77,1% in PUR, 51,2% in MXL and 61,5% in MEX; all three samples have the lowest African one (4-6%). Different studies about population admixture in Mexico showed different contributions. In the central and northern regions, Native American contribution goes from 32% to 69% Native-American while African is usually less than 7% (Martinez-Fierro *et al.*, 2009; Salzano and Sans, 2014). A comprehensive analysis by Rubi-Castellanos *et al.* (2009) in 10 Mexican regions shows somehow different results, presenting higher African contributions in some regions as Nueva Leon (18,5%), Veracruz (17,2%), and Jalisco and Campeche (15,9%).

The ancestry analysis by region evidenced a different result in each one of the 10 regions. While in some genes the Asian contribution (as a proxy of Native American) predominates in all the admixed samples (*MLH1* and *MSH6*), in the 16q22.1 region the European contribution prevails. However, in most of the regions, the predominant ancestry is not the same for all samples. In *MSH2*, the European contribution is predominant except in the ASW in which the African is the greatest. This exposes a different situation for each population and for each genomic region and outlines the importance of considering the local ancestry complementary to the global ancestry when performing association analysis in order to avoid spurious associations.

A similar conclusion can be drawn by taking into account the genetic variation analyses. The heterozygosity values showed very dissimilar ancestral contribution by population and by region. Only in one of the regions considered the highest and lowest mean values of heterozygosity were detected in one parental population (*MSH6*), being in most of the cases the highest mean value found in the African samples (all except *MUTHY* and *MSH6*). And finally, in four cases (*APC, BRAF, MUTHY* and *PMS2*), the lowest mean values were found in two admixed populations (PEL and MXL).

Both, in ancestry and in genetic variation analyses, the Native American contribution in Peruvian and Mexican samples is the highest, and consequently, it is possible to presuppose that the genetic variation patterns could be more closely related to Native Americans than in other Latin American populations. This is reflected in the *MSH2* gene heterozygosity values, as well as for haplotypic blocks of 5-10 kb, but not for the rest of the performed analyses. The non-expected values can be explained by different factors, like comparisons with Asian samples, instead of Native American samples. Moreover, some differences between the two Mexican samples were shown. The Mexican (MXL) sample was recruited in Los Angeles, California, and consequently, it can better be compared with Mexican Americans.

The MEX corresponds to the capital city, composed of subjects from the centre of the country, and Monterrey and Torreon, represented by subjects of northern parts of the country. There is also a difference of 10% of Native contribution, being greater in MEX than in MXL. Another crucial difference is the size of the samples (76 *versus* 831, respectively). This fact is not minor, as bigger samples may uncover heterogeneities due to subestructuration. Then, variation in different parameters can be explained because of that, as the apparent presence of variation in heterozygosity at 3’ and synonymous not found in MXL but in MEX in *MSH2* gene (Figure 2), or having more longer blocks shown in MEX sample (Figure 4). Also, differences between Mexican samples can be related to the coverage of the DNA analysis, being low for in MXL and high for MEX. It has been demonstrated by Ros-Freixedes *et al.* (2018) that low coverage can generate bias towards the detection of SNPs, showing that concordance with 10X coverage was 90,5% for genotypes and 95,2% for alleles, while with high coverage those values increased to 99,7 and 99,9%, respectively.

The size of blocks supports that admixed populations have higher values of linkage disequilibrium that lead to a specific pattern of haplotypic structures. For example, PUR showed the higher values of European ancestry but despite that, its heterozygosity values are close to EUR for *BRAF* and *MSH2,* but not for *APC* or for haplotypes, where PUR are more similar to the other admixed samples.

Besides African and African-derived populations showed smaller blocks than the other populations, it is necessary to note that all populations analyzed here show a broad range of small blocks indicating little recombination in the regions, most genes, studied. As Gabriel *et al.* (2002) have demonstrated, African and African-American populations have around half of the genome concentrated in blocks of 22 kb or larger. Here we showed an intermediate situation in the Latin American population, despite some differences depending on the degree of admixture (and the origin of the genetic contributions) and the chromosomal region analyzed.

Two facts can be highlighted: 1) several evolutionary forces- not only genetic flow- act on genetic variability; and 2) each region analyzed has special behavior when genetic variation is analyzed, despite all genes and chromosomal regions analyzed.

Related to the first, our data suggest that the patterns of ancestry and variability appear in certain genomic regions and under certain circumstances, but not in others. Different microevolutive forces such as selection, genetic drift, and eventually recombination, conversion and hitchhiking are probably present (Maynard Smith and Haigh, 1974). Moreover, the evolutionary processes act on genetic regions and genes, being selection (positive or negative) the most important, followed by others as mutations (Salzano, 2005). Besides, genetic flow is related to different migrations in the history of the involved populations that generated differences in populations and subpopulations (Stumpf and Goldstein, 2003; Choudhry *et al.*, 2006). Consequently, a deeper study taking into account historical and demographic scenarios as well as genetic variability is required before trying to make inferences.

Related to the second, the 10 analyzed regions were detected as associated with CRC in European populations (Kinzler *et al.*, 1991; Aaltonen *et al.*, 2007; Carvajal-Carmona *et al.*, 2011). Interestingly, when these regions were considered in the MEX sample when analyzing CRC in controls and patients, none of these genes showed association with the disease; only the 16q22.1 region was detected as associated (unpublished data). We would like to emphasize that our results suggest that not only global ancestry analysis is important when studying the association of genomic regions to a complex disease in admixed populations, but also regional ancestry analysis is advisable to be performed in order to detect an imbalance of ancestral contribution between cases and controls. Otherwise, associations might be the result of the mentioned imbalance rather than the possible implication of that region in the disease considered.

Several authors (among others, Tishkoff and Verrelli, 2003a,b; González Burchard *et al.*, 2005; Coop *et al.*, 2009) have pointed out the importance of evolutionary factors (such as admixture) to understand the genomic structure of populations. Our data support that each population history and each genomic region needs to be studied independently. Consequently, we emphasize the importance of a prospective analysis of ancestral characteristics of the populations to be studied, especially when dealing with the admixed Latin American populations where the di or tri-parental admix model is the most suitable.

Finally, this study strongly suggests the necessity of developing statistical methods to deal with di or tri-hybrid populations. It is also necessary to carefully analyze the different historical and demographic scenarios of each particular population to avoid generalizations, since, considering Latin America as a whole, is more theoretical than real.
